# Alterations in Vagal Tone Are Associated with Changes in the Gut Microbiota of Adults with Anxiety and Depression Symptoms: Analysis of Fecal Metabolite Profiles

**DOI:** 10.3390/metabo14080450

**Published:** 2024-08-15

**Authors:** Laura Pasqualette, Tatiana Kelly da Silva Fidalgo, Liana Bastos Freitas-Fernandes, Gabriela Guerra Leal Souza, Luís Aureliano Imbiriba, Leandro Araujo Lobo, Eliane Volchan, Regina Maria Cavalcanti Pilotto Domingues, Ana Paula Valente, Karla Rodrigues Miranda

**Affiliations:** 1 Instituto de Microbiologia Paulo de Góes, Universidade Federal do Rio de Janeiro, Rio de Janeiro 21941-902, Brazil; 2 Developmental and Educational Psychology, University of Bremen, 28359 Bremen, Germany; 3 Pediatric Dentistry, Department of Preventive and Community Dentistry, State University of Rio de Janeiro, Rio de Janeiro 20551-030, Brazil; 4 National Centre of Nuclear Magnetic Resonance/CENABIO, Medical Biochemistry, Federal University of Rio de Janeiro, Rio de Janeiro 21941-902, Brazil; 5 Laboratory of Psychophysiology, Department of Biological Sciences, Federal University of Ouro Preto, Ouro Preto 35400-000, Brazil; 6 School of Physical Education and Sports, Federal University of Rio de Janeiro, Rio de Janeiro 21941-599, Brazil; 7 Institute of Biophysics Carlos Chagas Filho, Federal University of Rio de Janeiro, Rio de Janeiro 21941-902, Brazil

**Keywords:** metabolomics, gut–brain axis, depression, anxiety, vagal tone, gut microbiota, amino acids, short-chain fatty acids

## Abstract

Accumulating evidence suggests that interactions between the brain and gut microbiota significantly impact brain function and mental health. In the present study, we aimed to investigate whether young, healthy adults without psychiatric diagnoses exhibit differences in metabolic stool and microbiota profiles based on depression/anxiety scores and heart rate variability (HRV) parameters. Untargeted nuclear magnetic resonance-based metabolomics was used to identify fecal metabolic profiles. Results were subjected to multivariate analysis through principal component analysis (PCA) and partial least squares discriminant analysis (PLS-DA), and the metabolites were identified through VIP score. Metabolites separating asymptomatic and symptomatic groups were acetate, valine, and glutamate, followed by sugar regions, glutamine, acetone, valerate, and acetoacetate. The main metabolites identified in high vagal tone (HVT) and low vagal tone (LVT) groups were acetate, valerate, and glutamate, followed by propionate and butyrate. In addition to the metabolites identified by the PLS-DA test, significant differences in aspartate, sarcosine, malate, and methionine were observed between the groups. Levels of acetoacetate were higher in both symptomatic and LVT groups. Valerate levels were significantly increased in the symptomatic group, while isovalerate, propionate, glutamate, and acetone levels were significantly increased in the LVT group. Furthermore, distinct abundance between groups was only confirmed for the Firmicutes phylum. Differences between participants with high and low vagal tone suggest that certain metabolites are involved in communication between the vagus nerve and the brain.

## 1. Introduction

In 2019, almost a billion people suffered from some mental disorder. Approximately 31% of people experienced symptoms of anxiety while the rate of depression was 29%. Factors such as financial worries and social pressure contribute to these high rates [1]. Concerning young adults, where globally the rates are 4%, academic pressure, and social media can increase impact on their mental health leading to feelings of stress, anxiety, and depression. Depression is an often disabling, lifelong illness that greatly increases the risk of suicidal behavior, affecting an estimated 346 million people worldwide [1]. However, current antidepressants are only fully effective in 30–40% of patients. Understanding the molecular and biochemical mechanisms underlying the disorder is necessary for improving prognosis, diagnosis, and current treatment options for patients with depression [2,3]. Anxiety disorders can develop alone or occur in conjunction with other disorders and are associated with the anticipation of future threats (i.e., anxiety) and emotional responses to real or perceived imminent threats (i.e., fear) [4]. Using validated tools for symptom assessment, several previous studies have demonstrated the co-occurrence of anxiety and depressive symptoms [5,6].

The National Institutes of Mental Health (NIMH) developed the Research Domain Criteria (RDoC), a multidisciplinary research framework for investigating mental illness. Due to the broad range of phenotypes among individuals with mental illness, the goal of the RDoC is to expand the criteria for mental illness diagnoses by linking molecular mechanisms to observable behavior. Although anxiety and depression exhibit high comorbidity, their severity is highly variable, highlighting the need to understand the physiological mechanisms underlying their development and chronicity [4,7].

Several metabolites are altered in depressive disorder, indicating involvement of cellular signaling molecules, cell membrane components, neurotransmitters, hormonal, inflammatory, and immune factors [8]. Metabolomics-based approaches have been used to identify biomarkers associated with depression and to analyze the effects of antidepressants in biofluids such as urine and plasma [3,8,9,10]. Studies investigating amino acid metabolism have reported that tryptophan levels are significantly decreased in the urine and plasma of patients with depression, relative to levels observed in control participants [3,11,12]. Because tryptophan is a precursor to many neurotransmitters, such decreases may reduce the biosynthesis of serotonin (5-HT) [13]. Furthermore, patients with depression exhibited significant increases in glutamic acid and phenylalanine levels, which were likely due to decreases in its conversion to tyrosine, in turn resulting in decreased levels of tyrosine metabolites (i.e., norepinephrine, epinephrine, and dopamine) [3,14]. A meta-analysis study revealed that metabolites from the tryptophan, tyrosine, and purine pathways exhibit altered expression patterns in individuals experiencing depressive symptoms [8]. The kynurenine pathway, which metabolizes tryptophan to kynurenine, is implicated as a critical factor, with lower plasma kynurenine levels associated with more severe depressive states. Additionally, decreasing acylcarnitine levels correlated with increased depression severity, suggesting a potential link to mitochondrial dysfunction [8]. Gut microbiota can contribute to changes in amino acids metabolism and theses alterations were associated with depression [15,16]. Consistent changes in the levels of several amino acids such increasing of glycine, alanine, citrate, and formate, and decreasing of phenylalanine, valine, aminoethanol, and Hippurate are associated with the behavior indices of depression. Furthermore, metabolic pathways such as biosynthesis of coenzyme Q and steroid, and metabolism of glycine–serine–threonine, tyrosine, and pyrimidine were found to be involved in the pathophysiology of depression [8].

Additional research groups have attempted to identify metabolic biomarkers for anxiety disorders in biofluids [17,18,19]. Previous studies have demonstrated that the gut microbiota–brain axis modulates nervous system development and activity [20]. Fermented milk products containing probiotics, which are known to alter the metabolism of the gut microbiota, have been associated with modulation of activity in several brain areas involved in sensory perception and emotion [21]. Emerging evidence has also revealed associations between gut microbiota and neurodevelopmental disorders [21,22]. Such studies have reported that the composition of the gut microbiome and the associated metabolic profiles differ between children with autism and healthy controls [23,24]. Furthermore, germ-free mice exhibit increased secretion of adrenocorticotropic hormone and corticosterone relative to their pathogen-free counterparts [25], resulting in a maladaptive response to stress and anxiety-like behavior [26].

Understanding the metabolites of otherwise healthy individuals exhibiting symptoms of anxiety and depression could provide useful insights, potentially facilitating early interventions before clinical diagnoses and medication. Furthermore, insights from metabolomic studies may lead to the development of personalized treatment options tailored to individual metabolic profiles [23].

Communication along the gut–brain axis may occur via the production of neurotransmitters, activation of immune cells and the enteric nervous system, activation of the hypothalamic–pituitary–adrenal (HPA) axis, or via the vagus nerve [27,28,29]. One practical method for studying autonomic activity is based on measurements of heart rate variability (HRV). HRV is conventionally accepted for describing variations in consecutive heartbeats and RR intervals (i.e., the period between R waves on electrocardiography (ECG)), which are controlled by vagal and sympathetic tone [30]. High HRV is considered healthy, as it indicates high flexibility to stress and environmental stimuli, whereas low HRV indicates low flexibility and can affect immunological functions, autoregulation, and psychosocial abilities [30]. Many studies have identified HRV parameters as biomarkers of psychiatric conditions, such as depression, anxiety, and posttraumatic stress disorder [31,32,33,34]. The Beck Depression Inventory is widely used to evaluate symptoms of depression [35], while the self-reported State-Trait Anxiety Inventory is used to identify the likelihood of developing anxiety throughout one’s lifetime [36].

Based on these previous findings, the present study aimed to investigate whether young, healthy adults without psychiatric diagnoses exhibit differences in metabolic stool and microbiota profiles based on depression/anxiety scores and HRV parameters.

## 2. Materials and Methods

### 2.1. Participants

Using the inclusion criteria specified by Pellissier et al. in 2014 [37], we recruited 18 healthy individuals (10 women and 8 men) between 18 and 25 years of age (mean: 22.5 ± 1.91 years) for the present study. All participants were free of major diseases, and none were taking medication that could have affected the autonomic nervous system. Participants were also required to be non-smokers and not consuming psychotropic drugs or taking antibiotics for at least 6 months. Moreover, 24 h prior to the experiment, participants were asked to refrain from using alcohol and/or illicit drugs. Two hours before the experiment, we advised participants not to ingest drinks containing caffeine or engage in strenuous physical activity. All participants provided written informed consent. The present study was approved by the Ethical Committee on Research of the University Hospital of the Universidade Federal do Rio de Janeiro (CEP/HUCFF/UFRJ: number 59014416.0.0000.5257).

### 2.2. Assessment of Depression and Anxiety

The Beck Depression Inventory II (BDI-II) [38] was used to evaluate depressive symptoms, while the State-Trait Anxiety Inventory (T-anxiety) (STAI-T) [39] was used to evaluate their propensity for developing anxiety.

### 2.3. Assessment of Heart Rate Variability

ECG recordings were acquired with a Biopac System (Goleta, CA, USA; model MP 150; analog-to-digital converter of 16 bits; sampling frequency: 1000 Hz), using a pair of Ag/AgCl surface electrodes (diameter: 8 mm) located at precordial derivation V5.

During data acquisition, participants were first invited to fill out a form concerning their personal characteristics, habits (i.e., age, sex, weight, height, drug usage, exercise habits), and diet (adaptation phase). They were seated comfortably and watched a 5-min video describing how to build a sandcastle (basal phase). We used an emotionally neutral video clip rather than a no-task baseline to assess HRV because minimally demanding tasks provide better estimates of resting cardiovascular activity [40].

The ECG signals were explored “off-line” using MATLAB R2007b (Mathworks, Natick, MA, USA). After a visual inspection of the ECG signal for peak correction and possible exclusion of individuals with noisy signals or arrhythmia, the time series of RR intervals were obtained from the R-wave peaks, which were identified using a threshold-based algorithm [41]. For temporal and spectral analysis, each signal was resampled at regular intervals via cubic spline interpolation (frequency: 2 Hz). The temporal parameter calculated from the RR interval time series was the root mean square of successive differences between RR intervals (RMSSD). For frequency-domain analyses of HRV, the power spectral density was calculated via fast Fourier transformation [42] of the main spectral components of the HRV signal: high frequency (HF; frequency range: 0.15–0.4 Hz) and low frequency (LF; frequency range: 0.04–0.15 Hz). Calculations were performed in accordance with the recommendations of the Task Force of the European Society of Cardiology and the North American Society of Pacing and Electrophysiology [43]. However, only the HF band and the RMSSD were used in the analysis, both of which represent the parasympathetic activity of the heart [30].

### 2.4. Fecal Sample Acquisition

Participants received a kit with a sterilized stool collector and a small bowl. Volunteers were asked to fill the bowl a maximum of two days prior to testing and to keep the sample in the refrigerator (4 °C) until delivery on experiment day. Samples were then stored at –80 °C until processing and analysis.

### 2.5. Evaluation of Microbiota Composition through Quantitative Real-Time Polymerase Chain Reaction (qRT-PCR)

DNA was extracted using a QIAamp^®^ DNA stool mini kit (Qiagen, Düsseldorf, Germany) and stored at –20 °C until analysis, following manufacturer instructions. DNA quantification was performed using a Qubit™ dsDNA BR Assay Kit (InvitrogenTM, Thermo Fischer Scientific, Waltham, MA, USA).

In accordance with the protocol described by Nunes et al. [44], we performed qRT-PCR for the three main phyla in the gut microbiota (Bacteroidota, Firmicutes, and Actinobacteria) using StepOnePlusTM (Life Technologies, Carlsbad, CA, USA).

### 2.6. Metabolite Extraction

Metabolites were extracted from fecal samples following methods described by Lamichhane et al. [45]. Phosphate-buffered saline (pH 7.1) with 5 mM azide was used for extraction, which was performed at a ratio of 1:2 (*w*/*v*). The final supernatant volume (1 mL) was stored at –80 °C until further analysis.

### 2.7. Metabolite Profiling Based on Nuclear Magnetic Resonance (NMR)

For the NMR analysis, 600 μL of supernatant was mixed with 60 μL of deuterium water (D2O; Cambridge Isotope Laboratories Inc., Tewksbury, MA, USA) and 1 μL of 20 mM 4,4-dimethyl-4-silapentane-1-sulfonic acid (DSS; Sigma-Aldrich, St. Louis, MO, USA). D2O was used as a lock to the magnetic field, while DSS was used as the chemical shift reference (δ = 0.00 ppm).

Spectra were obtained using a 500 MHz NMR spectrometer (Bruker Biospin, Rheinstetten, Germany) at 25 °C. We used the Carr-Purcell-Meiboom-Gill (CPMG) pulse sequence for the 1H spectrum with 512 scans. To confirm the identity of metabolites and assess any ambiguities, we also acquired 1H−1H total correlated spectroscopy (TOCSY) data using the following acquisition parameters: mixing time: 70 ms; 4.096 × 512 points [45]. Resonance assignments were made based on CPMG and 1H-1H-TOCSY experiments and confirmed using the Human Metabolome database (http://www.hmdb.ca/; accessed on 30 May 2024) [46].

### 2.8. Data and Statistical Analysis

The Grubbs test was employed to exclude extreme values, followed by the D’Agostino–Pearson and Shapiro–Wilk tests to assess the normality of scale scores, HRV parameters, and metabolite data. All parameters were considered to exhibit a normal distribution (*p* > 0.05). *t*-tests and N − 1 two-proportion tests were performed to examine differences between the groups. All analyses were performed using GraphPad Prism^®^ 6 (GraphPad Software, La Jolla, CA, USA).

#### 2.8.1. Depression and Anxiety

Because BDI-II and STAI-T scores were highly correlated, two participants without comorbid anxiety and depression were excluded from the final analysis (n = 16), to facilitate group categorization. Those with scores above the median on both scales were selected for the symptomatic group (presenting high symptoms), while those with scores below the median were selected for the asymptomatic group (presenting low symptoms) (BDI-II: 11.5 [1–34] and STAI-T: 44.5 [23–67]) [35,36,47,48]. The median division for the BDI-II was representative of the standard score: Individuals with scores of 11 or more were considered to have depressive symptoms, while those with lower scores were not. For the STAI-T, participants below the median division of 44.5 included those with low and low intermediary anxiety, while those above the median were considered to have high or high intermediary anxiety.

#### 2.8.2. Heart Rate Variability (HRV)

The 18 participants were split into groups according to mean RMSSD values (43.1 ± 17.7) [49,50]. As RMSSD values are considered representative of vagal tone [49], those with values above and below the mean were categorized into the high vagal tone (HVT) and low vagal tone (LVT) groups.

#### 2.8.3. Microbiota Composition

Parametric *t*-tests were used to detect differences in phyla abundance between groups. *p* < 0.05 was considered significant [44].

#### 2.8.4. Metabolome Analysis

We analyzed the CPMG spectra with water suppression using excitation sculpting with gradients and a T2 filter using AMIX software (version 3.9.15, Bruker Biospin, Ettlingen, Germany). To avoid edge effects, all spectra were overlaid for evaluation using Topspin (Bruker Biospin). Those that could not be corrected for phase and baseline were excluded from the analysis.

Each NMR spectrum was analyzed by integrating bucket size regions of 0.03 ppm and excluding water regions (5.12–4.5 ppm). The regions 0.664–0.742, 1.33–1.41, 1.67–1.73, 2.00–2.07, 2.39–2.51, 2.85–2.89, 2.92–2.94, 2.99–3.06, 3.00–3.04, 3.21–3.27, 3.51–3.56, 6.69–6.91, and 9.12–9.28 were also excluded due to edge effects. The datasets were stored in a matrix with the rows and columns representing the fecal samples and chemical shifts (157 buckets), respectively. We used DSS as the internal reference.

Data were analyzed following methods described by de Oliveira et al. [51]. The spectra were normalized by the sum of intensities and Pareto scaling before applying multivariate analysis using Partial Least Squares Discriminant Analysis (PLS-DA) and Orthogonal PLS-DA using Metaboanalyst 4.0 software (www.metaboanalyst.ca; accessed on 30 July 2022). Accuracy (ACC) was assessed through a cross-group assessment. After, we identified the main metabolites responsible for the distinction between the groups through variable importance in projection (VIP) scores that were obtained based on multivariate analysis (PLS-DA) for each comparison, and the corresponding metabolites were analyzed using peak intensity and univariate statistical analyses. Complementary, all buckets were subjected to univariate parametric *t*-tests for comparisons between groups. Furthermore, Pearson’s correlation coefficients and linear regression analyses were performed for all parameters. The level of statistical significance was set at *p* < 0.05.

After performing statistical analysis, the metabolites were identified for each bucket region that presented statistical significance in multivariate or univariate analysis. The identification strategy was based on the CPMG, TOCSY, and other experiments available in the Open Science Framework data repository available on https://doi.org/10.17605/OSF.IO/UVF4Y; accessed on 30 July 2024). The identification of metabolites was based on the “Human Metabolome Database” assignment references (http://www.hmdb.ca/; accessed on 30 May 2024). It adopted an untargeted metabolomics approach; therefore, our strategy was to identify the metabolites that presented statistical importance for the referred system, following our previous study [51].

## 3. Results

### 3.1. Main Characteristics of Groups

Table 1 depicts the main characteristics of each group concerning exercise, diet, BMI, and age. In addition to the expected differences in scale scores and HRV parameters, we observed a significant difference in the practice of exercise between the symptomatic and asymptomatic groups.

### 3.2. Microbiota Analysis

The symptomatic group exhibited a significantly higher abundance of Firmicutes than the asymptomatic group (*p* = 0.015; Figure S1). However, no significant differences in the abundance of Bacteroidota or Actinobacteria were observed.

### 3.3. Metabolomics Analysis

**Figure 1:**
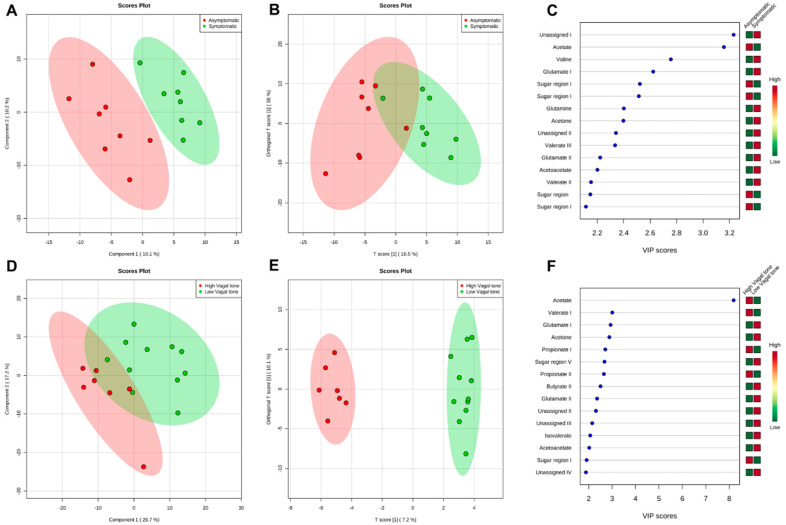
Representative of group separation and the loading factor for each separation. (**A**) PLS−DA of fecal samples from asymptomatic (red) and symptomatic (green) groups; (**B**) O−PLS−DA test of fecal samples from asymptomatic (red) and symptomatic (green) groups; (**C**) Loading factor comparing asymptomatic (red) and symptomatic (green) groups; (**D**) PLS−DA of fecal samples from High Vagal Tone (red) and Low Vagal Tone (green) groups; (**E**) O−PLS−DA test of fecal samples from High Vagal Tone (red) and Low Vagal Tone (green) groups; (**F**) Loading factor comparing High Vagal Tone (red) and Low Vagal Tone (green) groups.

Metabolomic differences were observed between the asymptomatic and symptomatic groups (5 PC; PC1 = 10.1% and PC2 = 10.2%), with an ACC of 0.67, R2 of 0.99, and AUC of 0.38 (sensitivity and sensibility = 0.63) (Figure 1A). Significant differences in orthogonal PLS-DA (OPLS-DA) were also observed (T score = 16.5% and OT score = 38%), with an R2X = 0.165, R2Y = 0.35, and Q2 = 0.137) (Figure 1B). Figure 1C displays the main metabolites separating the groups, based on multivariate analysis through VIP scores. The primary metabolites exhibiting such differences were acetate, valine, and glutamate, followed by sugar regions, glutamine, acetone, valerate, and acetoacetate.

VIP scores also showed significant differences between the HVT and LVT groups (5 PC; PC1 = 26.7% and PC2 = 17.2%), with an ACC of 0.66, R2 of 0.99, and an AUC of 0.67 (sensitivity = 1.0; sensibility = 0.45) (Figure 1D). The OPLS-DA test (T score = 7.2% and OT score = 10.1%) yielded the following values for p1: R2X = 0.07, R2Y = 0.6, and Q2 = 0.09. For o1, the following values were observed: R2X = 0.32, R2Y = 0.28, and Q2 = 0.19 (Figure 1E). The main metabolites identified were acetate, valerate, and glutamate (Figure 1F), followed by other short-chain fatty acids (SCFAs), such as propionate and butyrate (see Table S1).

We further evaluated metabolite intensity via a parametric univariate analysis. In addition to the metabolites identified by the PLS-DA test, significant differences in aspartate, sarcosine, malate, and methionine were observed between the groups (Table S2). Significant increases in acetoacetate levels were observed in both symptomatic and LVT groups (*p* = 0.02 and *p* = 0.006, respectively). Valerate levels were significantly increased in the symptomatic group (1.31–1.34 ppm; *p* = 0.04), while isovalerate, propionate, glutamate, and acetone levels were significantly increased in the LVT group. Our data were consistent with the changes observed in specific metabolic pathways.

**Figure 2:**
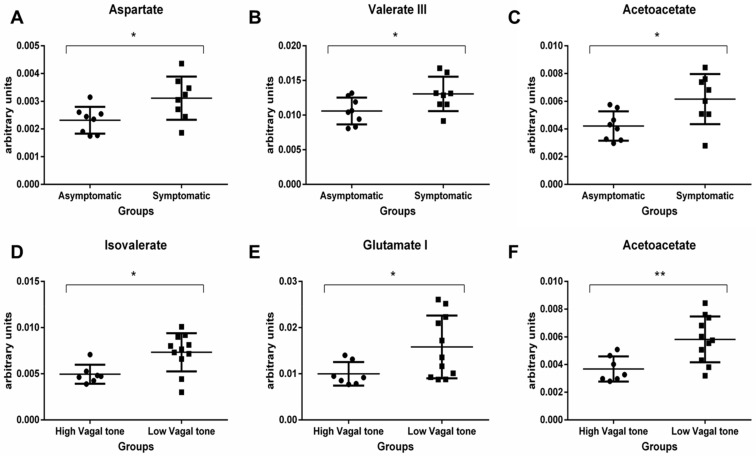
Metabolic pathways and their impact in the fecal metabolic profile changes, provided by Metaboanalyst 4.0. (**A**) Aspartate (*p* = 0.04), (**B**) Valerate (*p* = 0.04), and (**C**) Acetoacetate (*p* = 0.02) levels were significantly increased in the symptomatic group. (**D**) Isovalerate (*p* = 0.04), (**E**) Glutamate (*p* = 0.04), and (**F**) Acetoacetate (*p* = 0.006) levels were significantly increased in the LVT group. * indicates *p* < 0.05 and ** indicates *p* < 0.01.

Figure 2 shows the main pathways affected, which included glutamine/glutamate metabolism, ketone body/alanine metabolism, and aspartate/glutamate metabolism.

Figures S2 and S3 detail the metabolite variability observed between the asymptomatic and symptomatic groups, and between the HVT and LVT groups.

Furthermore, Pearson correlation analysis demonstrated positive correlations between RMSSD and HF components (r = 0.86; *p* < 0.001), and between BDI-II and STAI-T scores (r = 0.82; *p* < 0.001). The combination of scale scores and each HRV parameter yielded no significant correlations. However, we observed negative correlations between RMSSD and acetoacetate (*p* = 0.02), methionine (*p* = 0.01), and acetone levels (*p* = 0.01). Acetone levels also exhibited a negative correlation with HF components (r = 0.51; *p* = 0.03). In addition, BDI-II scores were positively correlated with valine levels (r = 0.53; *p* = 0.02), while STAI-T scores were positively correlated with acetoacetate levels (r = 0.58, *p* = 0.01) (Table 2). Similar results were obtained via linear regression analysis (Table S3).

## 4. Discussion

The present study aimed to explore potential differences in the metabolic and bacterial profiles of fecal samples from healthy participants, and whether such variances were associated with depressive symptoms, the propensity for anxiety, and vagal tone. Indeed, we observed significant differences in the metabolic profiles of each group, corroborating the findings of previous studies, which have reported that patients with depressive and stress-related disorders exhibit alterations in fecal metabolic composition [52,53,54,55]. Nevertheless, a key limitation of the current study was the small sample size. We also performed multiple comparisons to explore each hypothesis thoroughly. We highlight that this analysis applied in this reduced sample size increases the complexity of interpretation and the risk of false positives results. However, we have carefully considered these implications and ensured that our conclusions are statistically supported. This comprehensive approach was essential to provide a robust assessment and address our research questions effectively, despite the increased complexity of the results. Our results should be interpreted with caution; however, they signal that the effects of depressive and anxiety traits in the metabolome and microbiome should be further explored in future studies with larger samples. We hypothesize that underlying biomarkers of psychological symptoms might be found in the digestive system, which could potentially be used by doctors to aid individuals at a higher risk of developing comorbidities as a consequence of metabolic changes caused by neuropsychological symptoms [15,23]. Despite the limitations, the current analyses revealed distinct metabolic differences between individuals with low versus high vagal tone, as well as between those with and without depression/anxiety symptoms. In conclusion, additional studies are required to determine how and at which disease stages these metabolites can influence psychological states, and the present findings may contribute as a steppingstone to the development of strategies for modulating the gut microbiome in individuals presenting depression and anxiety symptoms.

Multivariate analysis using the VIP score approach revealed key metabolite differences between asymptomatic and symptomatic groups, including acetate, valine, glutamate, sugar regions, glutamine, acetone, valerate, and acetoacetate. Similarly, the high and low vagal tone groups exhibited distinct metabolite profiles, with acetate, valerate, glutamate, propionate, and butyrate as the main discriminators. Even with the limited sample size, we carried out the univariate analysis which elucidated significant differences in aspartate, sarcosine, malate, and methionine between the groups. Notably, acetoacetate levels were significantly elevated in both the symptomatic and low vagal tone cohorts. Additionally, the symptomatic group displayed increased valerate, while the low vagal tone group exhibited higher levels of isovalerate, propionate, glutamate, and acetone.

### 4.1. Short-Chain Fatty Acids (SCFAs)

Fiber polysaccharides escape digestion in the upper gastrointestinal tract and are metabolized by anaerobic bacteria in the hindgut. The major end products of this fermentative process are SCFAs, of which acetate, propionate, and butyrate account for 83%. The remaining end products include valerate, isobutyrate, and isovalerate [56,57]. As SCFAs have direct access to the brain, they may interfere with neurotransmission. Previous studies have indicated that SCFAs are associated with immunomodulation, neuromodulation, energy metabolism, and maintenance of the intestinal mucosal barrier [57].

Propionate is involved in gluconeogenesis, lipogenesis, and appetite regulation [58]. Acetate—the most abundant metabolite in the plasma and the only SCFA capable of crossing the blood–brain barrier (BBB)—has been identified as an appetite regulator and a key component of energy metabolism [57,59]. Although less studied than acetate, valerate has been associated with increased richness of the gut microbiota as well as obesity [60,61]. Interestingly, previous studies have reported that fecal valerate is decreased in individuals with high adhesion to the Mediterranean diet, whereas the opposite has been observed for acetate [62].

In the present study, propionate and acetate were present at higher levels in individuals with HVT, whereas butyrate and isovalerate levels were lower. In contrast, the LVT group exhibited higher levels of isovalerate, propionate, and valerate. Participants in the asymptomatic group also exhibited higher concentrations of acetate than the symptomatic group, who also exhibited increased levels of valerate, butyrate, and propionate. Szczesniak et al. [63] associated the increase in isovalerate with the detection of Faecalibacterium, Alistipes, Ruminococcus, and Ocillibacter in the microbiota of patients with depression. Interestingly, except for Alistipes of the Bacteroidota phylum, those genera belong to the Firmicutes phylum, which was more abundant in the symptomatic group. Zheng et al. [64] demonstrated that fecal samples from patients with major depressive disorder differed from those from healthy controls, exhibiting significant increases and decreases in some members of Firmicutes, respectively.

Butyrate is recognized (i) as an energy source for colonocytes, (ii) as a histone deacetylase (HDAC) inhibitor, (iii) for its anti-inflammatory properties, and (iv) for its role in the regulation of energy metabolism [65]. Some studies have demonstrated that treatment with butyrate can improve BBB permeability and integrity in mice [66] and ameliorate symptoms of depressive-like behavior [67]. In the present study, some members of Firmicutes may have been responsible for alterations in isovalerate and butyrate levels [65].

In a study involving 113 Belgian children ranging in age from 8 to 16 years, Michels et al. [54] observed that emotional problems were significantly associated with higher levels of butyrate, valerate, isovalerate, and isobutyrate. Higher parasympathetic activity was also associated with lower valerate levels. While the previous authors reported that valerate levels were correlated with LVT activity, we observed significant increases in valerate in the HVT group of the present study. This contradictory result is interesting when one considers the anti-inflammatory properties of butyrate. Increases in butyrate levels may therefore exert protective effects against inflammation, although previous studies have noted that the benefits of butyrate do not occur at physiological levels, but rather at high doses [65].

### 4.2. Ketone Bodies

Interestingly, we observed increased acetoacetate and acetone levels in symptomatic individuals and those with LVT. Ketone bodies, which also include β-hydroxybutyrate, are the main energy source in the hypoglycemic state [68,69]. Several previous studies have investigated the benefits of ketone bodies for patients with neural and metabolic diseases [69,70,71]. In a study by Ari et al. [72], ketone supplementation diminished anxiety symptoms in rats and increased blood levels of β-hydroxybutyrate, in contrast to our results. However, the differences in the biofluid analyzed may explain the contradictory findings. Sleiman et al. [73] demonstrated that exercise induces the production of brain-derived neurotrophic factor (BDNF) in the brain via β-hydroxybutyrate, which alleviates symptoms of depression and anxiety. In our study, exercise was only a significant factor between the symptomatic and asymptomatic groups, supporting the notion that exercise aids in the treatment and prevention of depression and anxiety [74]. Taken together, the accumulated evidence indicates that this effect may be mediated by the gut microbiota and gut metabolome.

Notably, the asymptomatic group and those with HVT exhibited higher concentrations corresponding to sugar regions. We hypothesize symptomatic individuals and those with LVT exhibit changes in energy metabolism that impact the conversion of acetoacetate to β-hydroxybutyrate. These metabolic alterations may be systemic, or they may be restricted to bacteria in the intestine and gut.

### 4.3. Amino Acids

We observed significant increases in glutamate and glutamine levels in the symptomatic and LVT groups, indicating changes in metabolic pathways that involve alanine and gamma-aminobutyric acid (GABA). The production of these two amino acids is essential for brain function, and both serve as energy sources for intestinal cells. Alterations in the brain profiles of these amino acids have been observed in patients with various disorders, including depression, anxiety, and schizophrenia [75].

Although glutamate is unable to cross the BBB, it is partly responsible for communication between bacteria and mucosal cells that possess glutamatergic, ionotropic, and metabotropic receptors [75]. In contrast, glutamine exhibits anti-inflammatory properties and protects the intestinal mucosal barrier, suggesting that glutamine supplementation can be used in the treatment of celiac disease and inflammatory bowel disease [76]. Indeed, Janik et al. [77] demonstrated that the probiotic Lactobacillus rhamnosus, a member of Firmicutes, increases levels of glutamate, glutamine, GABA, and N-acetyl aspartate in the brain after 2- and 4-week treatment in male mice. Thus, the observed changes in Firmicutes abundance may have been associated with increases in glutamate and glutamine production in the symptomatic and LVT groups.

In the present study, we observed higher levels of valine in fecal samples from the symptomatic group than in those from the asymptomatic group. Valine is a branched-chain amino acid acquired through a diet that can cross the BBB [78]. Baranyi et al. [79] reported that plasma levels of valine, leucine, and isoleucine are decreased in patients with depression and that valine levels are negatively correlated with BDI-II and Hamilton Depression Rating Scale scores. In contrast, we observed that valine levels were positively correlated with BDI-II scores in the present study, potentially due to differences in the biofluid analyzed.

Aspartate and malate levels were increased in the symptomatic and LVT groups [80]. Both aspartate and malate participate in energy metabolism. Alterations in energy metabolism and mitochondria are associated not only with depression and anxiety but also with schizophrenia [81].

Levels of sarcosine, an amino acid found in muscles and other organs, were increased in the symptomatic group of the present study. Sarcosine is an intermediary compound of choline/glycine metabolism and is also involved in methionine metabolism [82]. Sarcosine increases glutamatergic neurotransmission in the brain by enhancing the availability of glycine in the synaptic cleft, since it acts as a competitive inhibitor of glycine transporter type I and a co-agonist of the NMDA receptor [82]. These properties have been associated with protective effects against depression and anxiety in both humans and mice [83,84]. However, no studies have investigated the influence of intestinal sarcosine metabolism on the gut microbiota, highlighting the need for further research regarding its role in healthy organisms.

We observed decreased levels of methionine in the HVT group. Methionine is essential for the synthesis of S-adenosylmethionine, an important methyl group donor for DNA, RNA, proteins, and lipids [85]. S-adenosylmethionine has also been studied as a possible treatment for depression and anxiety [86].

Despite our findings, further studies are required to determine the precise roles of these metabolites within the intestine and systemically. Future studies should address not only their levels in healthy individuals and patients but also their roles in communication among bacteria and host cells. Although we could not quantitatively control for diet and exercise, these factors did not represent potential influences on the alterations observed in the present study, which provides insight into metabolites that may be associated with depression and the propensity for anxiety. Some studies suggest that the gut microbiota and intestinal metabolites communicate with the brain via the vagus nerve [29,87,88]. Therefore, future studies should investigate the association between vagal tone and fecal metabolites.

## 5. Conclusions

Demonstrated in this research is the distinct abundance of the Firmicutes phylum between groups. In addition, based on the differences between participants with high and low vagal tone, it is suggested that some metabolites are involved in communication between the vagus nerve and the brain. Considering the limitation of the present study, these results must be interpreted with caution. Even though our findings are promising, further research is required to determine how and why such metabolite alterations occur in the gut of individuals with depression and anxiety and/or low vagal tone.

## Figures and Tables

**Table 1:** Characteristics of each group. ** The group had a statistical difference in an N − 1 Two Proportion Test (*p* < 0.003). *** The group differed statistically according to unpaired non-parametric *t*-test (*p* < 0.0005).

	Asymptomatic (n = 8)	Symptomatic (n = 8)	High Vagal Tone (n = 11)	Low Vagal Tone (n = 7)
Gender (M:F)	5:3	2:6	3:4	5:6
Age ± SD	23 ± 1.8	22.3 ± 1.8	21.7 ± 1.8	23.1 ± 1.8
BMI ± SD	24.3 ± 5.0	23.0 ± 1.9	22.4 ± 2.2	24.5 ± 4.3
Exercise (Y:N)	7:1 **	1:7 **	5:2	4:7
Eating habits (O:V)	8:0	6:2	5:2	10:1
HF ± SD	1008.1 ± 875.8	1023.6 ± 915.5	2036.1 ± 910.9 ***	490.7 ± 405.4 ***
RMSSD ± SD	43.6 ± 13.3	38.2 ± 13.6	60.6 ± 13.8 ***	31.9 ± 8.2 ***
BDI-II Scores ± SD	5.8 ± 3.8 ***	22.3 ± 7.0 ***	10.8 ± 11.1	15.4 ± 8.6
STAI-T Scores ± SD	33.8 ± 7.1 ***	53.2 ± 8.2 ***	40.1 ± 14.8	46.4 ± 9.2

To describe different factors, it was used the Mean ± Standard Deviation (SD). M:F = Male/Female; Y:N = Yes (exercise)/No (do not exercise); O:V = Omnivorous/Vegetarian; HF: high frequency; RMSSD: RR intervals; BMI: Body Mass Index; BDI-II: Beck Depression Inventory II; STAI-T: State-Trait Anxiety Inventory.

**Table 2:** Pearson correlation among physiological/psychological parameters and metabolites. Bold number indicate statistical differences (*p* < 0.05).

	RMSSD	BDI-II	STAI-T	HF
RMSSD		−0.24	−0.09	**0.86**
BDI-II	−0.24		**0.82**	0.004
STAI-T	−0.09	**0.82**		0.19
HF	**0.86**	0.004	0.19	
Butyrate (HMDB0000883)	−0.05	0.29	0.32	−0.04
Acetate (HMDB00042)	0.14	−0.05	−0.27	0.04
Propionate (HMDB00237)	0.26	0.10	−0.17	0.10
Glutamate (HMDB00148)	−0.45	0.14	0.25	−0.26
Glutamine (HMDB0003423)	−0.09	0.01	0.22	−0.02
Acetoacetate (HMDB0304256)	**−0.53**	0.44	**0.58**	−0.45
Valerate (HMDB00892)	0.30	0.25	−0.03	0.20
Aspartate (HMDB00191)	−0.37	0.43	0.39	−0.33
Valine (HMDB0000883)	−0.21	**0.53**	0.41	−0.01
Isovalerate (HMDB0000718)	−0.39	0.00	0.21	−0.42
Methionine (HMDB00696)	**−0.58**	0.24	0.19	−0.46
Malate (HMDB0031518)	−0.47	0.46	0.32	−0.38
Sarcosine (HMDB00271)	−0.12	0.32	0.41	−0.13
Acetone (HMDB01659)	**−0.58**	0.43	0.37	**−0.51**

RMSSD: RR intervals; BDI-II: Beck Depression Inventory II; STAI-T: State-Trait Anxiety Inventory; HF: high frequency.

## Data Availability

The data are available at https://doi.org/10.17605/OSF.IO/3D7PB (accessed on 30 July 2024) and https://doi.org/10.17605/OSF.IO/UVF4Y (accessed on 30 July 2024).

## Supplementary Materials

The following supporting information can be downloaded at: https://www.mdpi.com/article/10.3390/metabo14080450/s1, Figure S1. Representative spectra of symptomatic (A) and asymptomatic (B) subjects, showing the NMR assignments for important metabolites. 1H 1D spectra were obtained at 500 MHz in a Bruker Advance spectrometer at 25 °C; Figure S2. Metabolic pathways and their impact in the fecal metabolic profile changes, provided by Metaboanalyst 4.0; Figure S3. Difference in relative abundance of the Actinobacteria, Bacteroidota, and Firmicutes phyla between symptomatic and asymptomatic groups. Only the Firmicutes phylum presented a statistically significant difference between groups (*p* = 0.015); Table S1. Performances indexes of the classifications performed with PLS-DA methods for fecal metabolites from Asymptomatic × Symptomatic and High Vagal tone × Low Vagal tone groups; Table S2. Metabolite intensities shown as the mean (arbitrary units), confidence interval, bucket region (ppm), and statistical analysis from multivariate analysis using the VIP score and parametric *t*-test univariate analysis (*p*< 0.05). NSD = No statistical difference in the *t*-test. Table S3. Linear regression among parameters and metabolites. Bold number indicate statistical differences (*p* < 0.05).
