# Supplementary material for: Alterations in Vagal Tone Are Associated with Changes in the Gut Microbiota of Adults with Anxiety and Depression Symptoms: Analysis of Fecal Metabolite Profiles
Source: Metabolites. 2024 Aug 15;14(8):450. doi: 10.3390/metabo14080450 (PMC11356408; doi:10.3390/metabo14080450)
Supplement: Supplementary file 1 [file metabolites-14-00450-s001.zip › metabolites-3108715-supplementary.pdf]

SUPPLEMENTARY MATERIAL

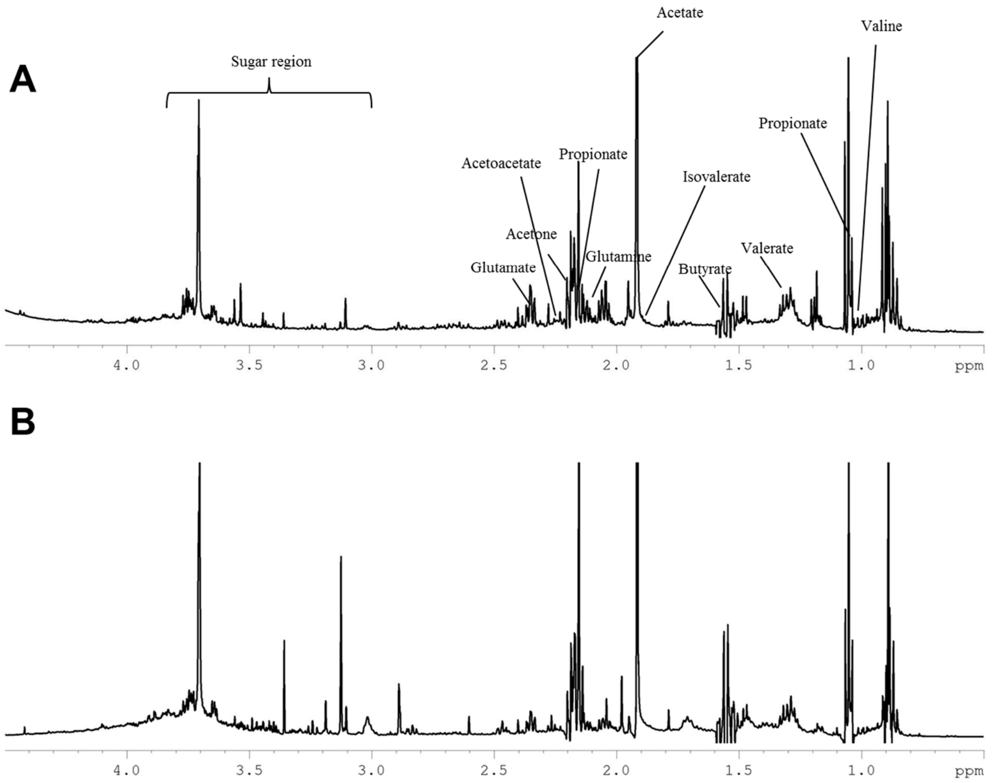

Figure S1. Representative spectra of symptomatic (**A**) and asymptomatic (**B**) subjects, showing the NMR assignments for important metabolites. ^{1}H 1D spectra were obtained at 500 MHz in a Bruker Advance spectrometer at 25ºC.

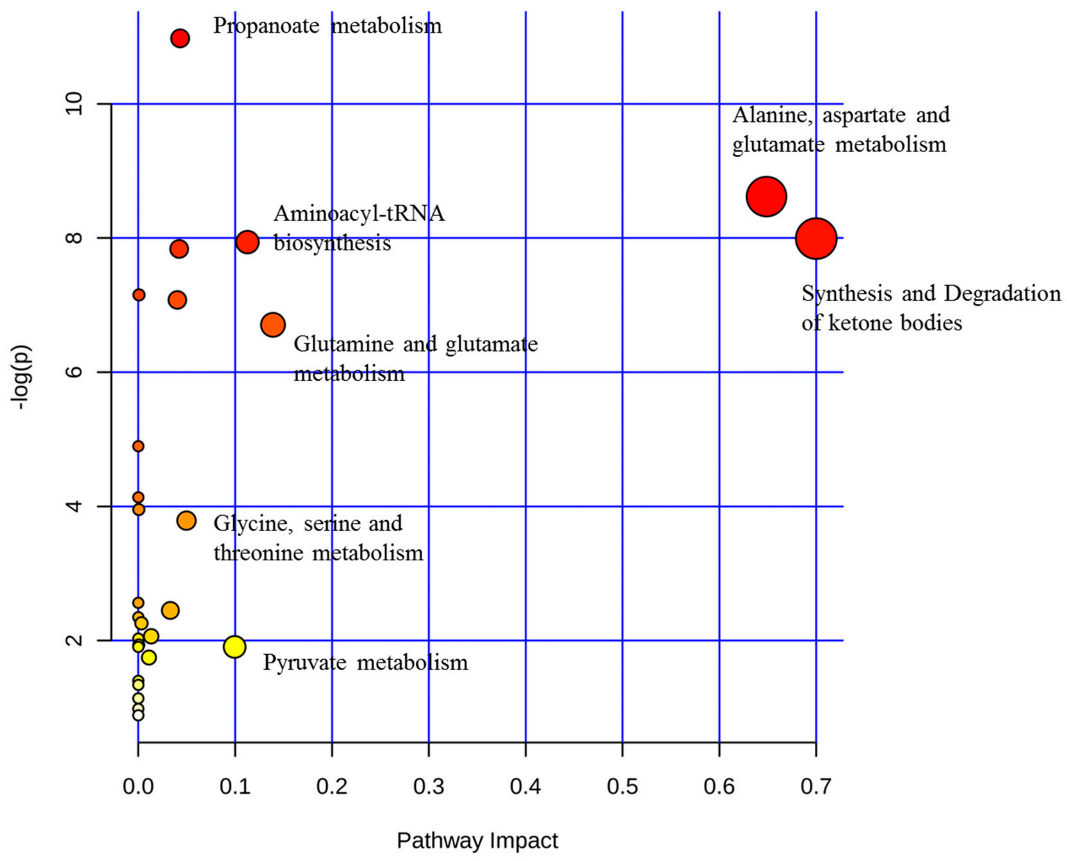

Figure S2. Metabolic pathways and their impact in the fecal metabolic profile changes, provided by Metaboanalyst 4.0.

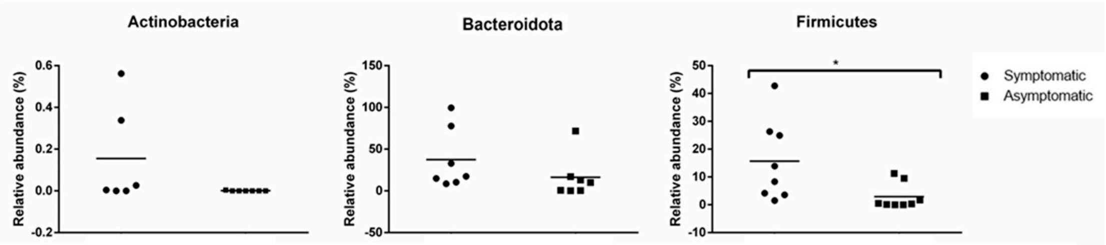

Figure S3. Difference in relative abundance of the Actinobacteria, Bacteroidota and Firmicutes phyla between symptomatic and asymptomatic groups. Only the Firmicutes phylum presented a statistically significant difference between groups ($p = 0.015$).

Table S1. Performances indexes of the classifications performed with PLS-DA methods for fecal metabolites from Asymptomatic x Symptomatic and High Vagal tone x Low Vagal tone groups.

**Model Performance**	**Asymptomatic x Symptomatic**	**High Vagal tone x Low Vagal tone**
ACC	0.68	0.66
R^2	0.99	0.99
Q^2	0.19	0.01
AUC	0.38	0.67
Sensitivity	0.63	1.00
Specificity	0.63	0.45

Table S2. Metabolite intensities shown as the mean (arbitrary units), confidence interval, bucket region (ppm) and statistical analysis from multivariate analysis using the VIP score and parametric t-test univariate analysis ($p < 0.05$). NSD = No statistical difference in the t-test.

Metabolites and IDs	ppm region	A Asymptomatic	B Symptomatic	C Low Vagal Tone	D High Vagal Tone	*p*-value
Acetate [1] (HMDB00042)	1.91	21.0×10^{-2} ($8.2 \times 10^{-2} – 36.5 \times 10^{-2}$)	19.1×10^{-2} ($14.3 \times 10^{-2} – 30.0 \times 10^{-2}$)	18.4×10^{-2} ($11.4 \times 10^{-2} – 30.0 \times 10^{-2}$)	24.2×10^{-2} ($8.2 \times 10^{-2} – 36.5 \times 10^{-2}$)	NSD
Acetoacetate [1,2] (HMDB0304256)	2.27	0.4×10^{-2} ($0.2 \times 10^{-2} – 0.5 \times 10^{-2}$)	0.6×10^{-2} ($0.2 \times 10^{-2} – 0.8 \times 10^{-2}$)	0.5×10^{-2} ($0.3 \times 10^{-2} – 0.8 \times 10^{-2}$)	0.3×10^{-2} ($0.2 \times 10^{-2} – 0.5 \times 10^{-2}$)	0.02^{AB} 0.006^{CD}
Acetone [1,2] (HMDB01659)	2.21	0.5×10^{-2} ($0.2 \times 10^{-2} – 0.9 \times 10^{-2}$)	0.8×10^{-2} ($0.1 \times 10^{-2} – 1.3 \times 10^{-2}$)	0.8×10^{-2} ($0.4 \times 10^{-2} – 1.3 \times 10^{-2}$)	0.4×10^{-2} ($0.1 \times 10^{-2} – 0.8 \times 10^{-2}$)	0.005^{CD}
Aspartate [2] (HMDB00191)	2.69	0.2×10^{-2} ($0.1 \times 10^{-2} – 0.3 \times 10^{-2}$)	0.3×10^{-2} ($0.2 \times 10^{-2} – 0.4 \times 10^{-2}$)	0.2×10^{-2} ($0.1 \times 10^{-2} – 0.4 \times 10^{-2}$)	0.2×10^{-2} ($0.1 \times 10^{-2} – 0.2 \times 10^{-2}$)	0.02^{AB} 0.04^{CD}
Butyrate II [1] (HMDB0000039)	1.55			0.6×10^{-2} ($0.0 \times 10^{-2} – 1.0 \times 10^{-2}$)	0.1×10^{-2} ($0.0 \times 10^{-2} – 0.4 \times 10^{-2}$)	NSD
Glutamine [1] (HMDB0003423)	2.12	1.1×10^{-2} ($0.8 \times 10^{-2} – 1.5 \times 10^{-2}$)	1.1×10^{-2} ($0.9 \times 10^{-2} – 2.3 \times 10^{-2}$)			NSD
Glutamate I [1,2] (HMDB00148)	2.33	1.2×10^{-2} ($0.7 \times 10^{-2} – 2.2 \times 10^{-2}$)	1.6×10^{-2} ($0.9 \times 10^{-2} – 2.6 \times 10^{-2}$)	1.5×10^{-2} ($0.8 \times 10^{-2} – 2.6 \times 10^{-2}$)	0.9×10^{-2} ($0.7 \times 10^{-2} – 1.4 \times 10^{-2}$)	0.04^{CD}
Glutamate II [1,2] (HMDB00148)	2.36	0.6×10^{-2} ($0.4 \times 10^{-2} – 1.0 \times 10^{-2}$)	0.8×10^{-2} ($0.4 \times 10^{-2} – 1.2 \times 10^{-2}$)	0.8×10^{-2} ($0.4 \times 10^{-2} – 1.2 \times 10^{-2}$)	0.5×10^{-2} ($0.4 \times 10^{-2} – 0.6 \times 10^{-2}$)	0.01^{CD}
Isovalerate [1,2] (HMDB0000718)	1.97			0.7×10^{-2} ($0.3 \times 10^{-2} – 1.0 \times 10^{-2}$)	0.4×10^{-2} ($0.3 \times 10^{-2} – 0.7 \times 10^{-2}$)	0.01^{CD}
Malate [2] (HMDB0031518)	2.66	0.3×10^{-2} ($0.2 \times 10^{-2} – 0.4 \times 10^{-2}$)	0.4×10^{-2} ($0.2 \times 10^{-2} – 0.5 \times 10^{-2}$)	0.4×10^{-2} ($0.2 \times 10^{-2} – 0.5 \times 10^{-2}$)	0.2×10^{-2} ($0.1 \times 10^{-2} – 0.3 \times 10^{-2}$)	0.04^{AB} 0.02^{CD}
Methionine [2] (HMDB00696)	2.63			0.4×10^{-2} ($0.1 \times 10^{-2} – 0.5 \times 10^{-2}$)	0.2×10^{-2} ($0.2 \times 10^{-2} – 0.3 \times 10^{-2}$)	0.01^{CD}
Propionate I [1] (HMDB00237)	1.04			4.0×10^{-2} (1.8×10^{-2} - 8.1×10^{-2})	4.8×10^{-2} ($3.4 \times 10^{-2} – 6.1 \times 10^{-2}$)	NSD
Propionate II [1] (HMDB00237)	2.15			3.4×10^{-2} ($1.3 \times 10^{-2} – 5.6 \times 10^{-2}$)	4.2×10^{-2} ($2.3 \times 10^{-2} – 6.0 \times 10^{-2}$)	NSD

Sarcosine [2] (HMDB00271)	2.72	0.2 x 10^{-2} (0.1 x 10^{-2} – 0.3 x 10^{-2})	0.3 x 10^{-2} (0.1 x 10^{-2} – 0.4 x 10^{-2})			0.04^{AB}
Sugar region I [1]	3.38	0.5 x 10^{-2} (0.2 x 10^{-2} – 1.8 x 10^{-2})	0.2 x 10^{-2} (0.2 x 10^{-2} – 0.3 x 10^{-2})			NSD
Sugar region II [1]	3.41	0.7 x 10^{-2} (0.2 x 10^{-2} – 2.4 x 10^{-2})	0.3 x 10^{-2} (0.2 x 10^{-2} – 0.4 x 10^{-2})			NSD
Sugar region III [1]	3.47	0.6 x 10^{-2} (0.2 x 10^{-2} – 1.7 x 10^{-2})	0.3 x 10^{-2} (0.2 x 10^{-2} – 0.5 x 10^{-2})	3.2 x 10^{-2} (1.0 x 10^{-2} – 11.2 x 10-12)	4.2 x 10^{-2} (0.7 x 10^{-2} –15.5 x 10^{-2})	NSD
Sugar region IV [1]	3.68	4.5 x 10^{-2} (1.1 x 10^{-2} – 6.2 x10^{-2})	3.4 x 10^{-2} (1.0 x 10^{-2} – 11.2 x 10^{-2})	2.1 x 10^{-2} (1.0 x 10^{-2} – 3.2 x 10^{-2})	1.6 x 10^{-2} (1.4 x 10^{-2} – 1.7 x 10^{-2})	NSD
Unassigned I[2]	1.07	0.1 x 10^{-2} (0.0 x 10^{-2} – 0.2 x10^{-2})	0.3 x 10^{-2} (0.1 x 10^{-2} – 0.9 x 10^{-2})			0.005^{AB}
Unassigned II[1,2]	1.94	1.0 x 10^{-2} (0.5 x 10^{-2} – 1.5 x 10^{-2})	1.3 x 10^{-2} (1.0 x 10^{-2} – 1.8 x 10^{-2})	1.3 x 10^{-2} (0.6 x 10^{-2} –1.8 x 10^{-2})	0.9 x 10^{-2} (0.5 x 10^{-2} – 1.3 x 10^{-2})	0.03^{AB} 0.04^{CD}
Unassigned III [1,2]	2.24			0.7 x 10^{-2} (0.4 x 10^{-2} – 1.0 x 10^{-2})	0.5 x 10^{-2} (0.2 x 10^{-2} – 0.9 x 10^{-2})	0.01^{CD}
Unassigned IV [1,2]	2.30			0.4 x 10^{-2} (0.3 x 10^{-2} – 0.6 x 10^{-2})	0.3 x 10^{-2} (0.2 x 10^{-2} – 0.4 x 10^{-2})	0.002^{CD}
Valerate I [1] (HMDB00892)	0.89			3.3 x 10^{-2} (1.5 x 10^{-2} – 7.0 x10^{-2})	4.3 x 10^{-2} (2.5 x 10^{-2} – 6.6 x 10^{-2})	NSD
Valerate II [1] (HMDB00892)	1.31	1.2 x 10^{-2} (0.8 x 10^{-2} – 1.5 x 10^{-2})	1.5 x 10^{-2} (0.9 x 10^{-2} – 2.0 x 10^{-2})			NSD
Valerate III [1,2] (HMDB00892)	1.34	1.0 x 10^{-2} (0.8 x 10^{-2} – 1.3 x 10^{-2})	1.3 x 10^{-2} (0.9 x 10^{-2} – 1.6 x 10^{-2})			0.04^{AB}
Valine [1] (HMDB0000883)	1.01	0.4 x 10^{-2} (0.0 x 10^{-2} – 0.8 x 10^{-2})	0.7 x 10^{-2} (0.4 x 10^{-2} – 1.9 x 10^{-2})			NSD

[1] Metabolites presenting a statistical difference in VIP score; [2] Metabolites presenting a statistical difference in parametric t-test univariate analysis.

Table S3. Linear regression among parameters and metabolites. Bold letters indicate statistical differences (p<0.05).

	**RMSSD**	**BDI-II**	**STAI-T**	**HF**
RMSSD		0.05	0.08	**0.74**
BDI-II	0.05		**0.67**	0.00
STAI-T	0.008	**0.67**		0.03
HF	**0.74**	0.00	0.03	
Butyrate	0.002	0.08	0.10	0.001
Acetate	0.02	0.002	0.07	0.001
Propionate	0.06	0.01	0.02	0.009
Glutamate	0.20	0.01	0.06	0.06
Glutamine	0.008	0,00	0.04	0.00
Acetoacetate	**0.29**	0.19	**0.33**	0.20
Valerate	0.09	0.06	0.001	0.04
Aspartate	0.13	0.18	0.15	0.10
Valine	0.04	**0.28**	0.16	0.00
Isovalerate	0.14	0.00	0.04	0.17
Methionine	**0.33**	0.05	0.03	0.21
Malate	0.22	0.21	0.10	0.14
Sarcosine	0.01	0.10	0.16	0.01
Acetone	**0.34**	0.18	0.13	**0.26**
